# GnRH antagonist rescue protocol combined with cabergoline versus cabergoline alone in the prevention of ovarian hyperstimulation syndrome: a randomized controlled trial

**DOI:** 10.1186/s13048-016-0237-8

**Published:** 2016-05-17

**Authors:** Usama M. Fouda, Ahmed M. Sayed, Hesham S. Elshaer, Bahaa Eldin M. Hammad, Mona M. Shaban, Khaled A. Elsetohy, Mohamed A. Youssef

**Affiliations:** Department of Obstetrics and Gynecology, Faculty of Medicine, Cairo University, Kasr Al-Ainy Hospital, Al-Saraya Street, Cairo, Egypt

**Keywords:** Cabergoline, IVF-ET, Ovarian hyperstimulation syndrome, GnRH antagonist

## Abstract

**Background:**

The aim of this study was to compare the efficacy of antagonist rescue protocol (replacing GnRH agonist with GnRH antagonist and reducing the dose of gonadotropins) combined with cabergoline versus cabergoline alone in the prevention of ovarian hyperstimulation syndrome (OHSS) in patients pretreated with GnRH agonist long protocol who were at high risk for OHSS.

**Methods:**

Two hundred and thirty six patients were randomized in a 1:1 ratio to the cabergoline group or the antagonist rescue combined with cabergoline group. Both groups received oral cabergoline (0.5 mg/day) for eight days beginning on the day of HCG administration. In the antagonist rescue combined with cabergoline group, when the leading follicle reached 16 mm, GnRH agonist (triptorelin) was replaced with GnRH antagonist (cetrorelix acetate) and the dose of HP-uFSH was reduced to 75 IU/day. HCG (5,000 IU/I.M) was administered when the serum estradiol level dropped below 3500 pg/ml. The study was open label and the outcome assessors (laboratory staff and the doctor who performed oocyte retrieval) were blind to treatment allocation.

**Results:**

The incidence of moderate/severe OHSS was significantly lower in the antagonist rescue combined with cabergoline group [5.08 % Vs 13.56 %, P value =0.025, OR = 0.342, 95 % CI, 0.129–0.906]. Four cycles were cancelled in the cabergoline group. There were no significant differences between the groups with respect to the number of retrieved oocytes, metaphase II oocytes, high quality embryos and fertilization rate. Moreover, the implantation and pregnancy rates were comparable between both groups.

**Conclusion:**

GnRH antagonist rescue protocol combined with cabergoline is more effective than cabergoline alone in the prevention of OHSS.

**Trial registration:**

Clinical trial.gov (NCT02461875).

## Background

Ovarian hyperstimulation syndrome (OHSS) is the most dangerous iatrogenic complication associated with ovarian stimulation. Cystic enlargement of the ovaries, increased capillary permeability and fluid shift from the intravascular space into the third space are the main characteristics of OHSS [1].

Several strategies have been proposed to prevent or minimize the intensity of OHSS. Cycle cancellation, withholding gonadotropins administration and postponing human chorionic gonadotropin (HCG) injection till HCG drops to a safe level (coasting),antagonist rescue protocol, embryos freezing, minimal stimulation IVF, in vitro maturation of oocytes, metformin administration in patients with polycystic ovary syndrome, natural cycle IVF, triggering ovulation with a reduced dose of HCG or with GnRH agonist in GnRH antagonists stimulation cycle and administration of GnRH antagonist after oocyte retrieval have all been used. Apart from cycle cancelation, none of these strategies have been effective in the complete prevention of OHSS [1, 2].

The primary pathophysiological feature of OHSS is increased capillary permeability, which results in fluid shift to the third space. It appears that the main cause of increased capillary permeability is the release of vasoactive-angiogenic substances (interleukins, tumor necrosis factor (TNF) α, endothelin and vascular endothelial growth factor (VEGF)) from the ovaries in response to exogenous or endogenous HCG [1, 2]. Moreover, several authors suggested that the circulating peripheral blood mononuclear cells may be involved in the pathophysiology of OHSS. In vitro studies, revealed that HCG stimulates the production of IL8 and VEGF by monocytes [3]. Kosaka et al. [4] observed that peripheral blood mononuclear cells of patients with OHSS produce excessive amounts of VEGF. Several studies have revealed that VEGF is the primary vasoactive-angiogenic substance responsible for the development of OHSS. VEGF stimulates neovascularization and increased capillary permeability by interacting with VEGF receptor 2 (VEGFR-2). Gonadotropins stimulate the expression of VEGF and VEGFR-2 mRNA in granulosa cells and HCG (exogenous or endogenous) accentuates this expression [5, 6].

Recent animal and human studies have revealed that dopamine agonists antagonize VEGF dependent vascular permeability by inhibiting VEGFR-2 phosphorylation and signaling. The use of cabergoline (a dopamine receptor-2 agonist) in the prevention of OHSS has been investigated in several studies. Three meta-analyses have revealed that cabergoline reduces the incidence of moderate/severe OHSS without affecting implantation, pregnancy and miscarriage rates [2, 6, 7].

Antagonist rescue protocol (replacing GnRH agonist with GnRH antagonist and reducing the dose of gonadotropins) is another effective strategy for preventing OHSS. Several studies have shown that the antagonist rescue protocol causes a rapid reduction in the estradiol level, prevents cycle cancelation, and reduces the risk of moderate/severe OHSS [8, 9].

Recently, Rollene et al. [10] reported the use of cabergoline and GnRH antagonist (ganirelix) in the outpatient treatment of four patients with OHSS. Cabergoline combined with ganirelix resulted in rapid resolution of the clinical symptoms of OHSS. None of the patients required hospitalization.

The aim of this study was to compare the efficacy of antagonist rescue protocol combined with cabergoline versus cabergoline alone in the prevention of ovarian hyperstimulation syndrome (OHSS) in patients pretreated with GnRH agonist long protocol who were at high risk for OHSS.

## Methods

During the period between March 2013 and December 2015, this prospective, two arm, assessor blinded, randomized controlled trial was conducted at the assisted conception unit of Aljazeera (Al Gazeera) hospital, Giza, Egypt. The study protocol followed the CONSORT guidelines for reporting parallel group randomised trials [11]. This study included 236 patients who were stimulated using the long luteal GnRH agonist protocol and at high risk for developing OHSS [have more than 20 follicles (90 % of them less than 14 mm in mean diameter) and serum estradiol ≥ 3000 pg/ml] [9]. The exclusion criteria were an age less than 18 years or more than 37 years, endometriosis, presence of submucous or intramural myoma of more than 5 cm, recurrent abortion and a previous IVF-ET cycle. Patients with lung fibrosis, swelling or inflammation around the heart or lung, hypertension, liver disease, heart valve disease, or an allergy to cabergoline or ergot derivatives were also excluded from the study. The ethics committee of the hospital approved the study protocol [N-2-2013] and the patients provided informed consent before randomization. The study was registered at clinical trials.gov [NCT02461875].

Patients were randomized to the cabergoline group and the antagonist rescue combined with cabergoline group using a computer generated randomization list (obtained from http://graphpad.com/quickcalcs/randomN1.cfm) and sequentially numbered sealed envelopes containing allocation information written on a card. The randomization list and sealed envelopes were prepared by a statistician who was not involved in the allocation process. The sequentially numbered envelopes were opened by the study nurse and the patients were informed of their assigned group. The laboratory staff and the doctor who performed oocyte retrieval (Fouda U.M) were blinded to the treatment received.

All patients recruited to this study were stimulated using the long luteal GnRH agonist protocol [12]. Triptorelin (Decapeptyl, Ipsen, Slough, United Kingdom) at a daily dose of 0.1 mg/S.C was started one week before anticipated menstruation. In PCOS patients with oligomenorrhea or amenorrhea, medroxyprogesterone acetate tablets (Provera Pfizer, Inc., New York, NY) (5 mg twice daily) were administered orally for 5 days to induce withdrawal bleeding. Following withdrawal bleeding, the patients received a 3-week course of combined oral contraception pills (Gynera, Bayer-Shering, Pharma-AG, Germany) from day 2 of menstruation. Triptorelin (0.1 mg/day) was started from day 20 of the cycle. After confirmation of pituitary down regulation (serum E_2_ levels less than 50 pg/ml and endometrial thickness less than 5 mm) highly purified urinary FSH(HP-uFSH) (Fostimon, IBSA, Switzerland) was administered. The starting gonadotropins dose was determined according to the age, antral follicle count, basal FSH and E_2_ levels and body mass index. Follicular development was monitored by transvaginal ultrasonography and serum estradiol. The starting dose of gonadotropins was not adjusted during stimulation.

In cabergoline group, cabergoline (Dostinex; Pfizer, Italy) at a daily dose of 0.5 mg was administered orally at bed time for 8 days starting on the day of HCG administration. HCG (Pregnyl; N.V. Organon, Oss, Holland) at a dose of 5,000 IU/I.M was administered when three or more follicles ≥ 18 mm in diameter were detected by ultrasound examination.

In antagonist rescue combined with cabergoline group, when the leading follicle reached 16 mm, HP-uFSH was continued at a daily dose of 75 IU until and including the day of HCG administration. Triptorelin was discontinued and GnRH antagonist (Cetrorelix acetate)(Cetrotide; Serono International S.A., Geneva, Switzerland) 0.25 mg/S.C was administered daily until and including the day of HCG administration. Serum estradiol was measured daily and HCG (5,000 IU/I.M) was administered when the serum estradiol level dropped below 3500 pg/ml. Moreover, cabergoline (0.5 mg/day) was administered orally at bed time for 8 days starting on the day of HCG administration.

Oocyte retrieval was performed under deep sedation 36 ± 2 h after HCG administration. Two or three embryos were transferred 3 days after oocyte retrieval. Pregnancy test was performed two weeks after embryo transfer. Transvaginal ultrasound was performed five weeks after embryo transfer to detect the presence of intrauterine gestational sac.

The luteal phase was supported with progesterone vaginal pessaries (Prontogest, Marcyrl, Egypt) 400 mg twice daily, starting on the day of oocyte retrieval until 12 weeks gestation or negative B-subunit HCG. The occurrence and severity of OHSS was defined according to the classification described by Golan et al. [13]. The detection of ascites by ultrasound examination in patients with features with mild OHSS (enlarged ovaries (5–12 cm), abdominal discomfort, nausea, vomiting and diarrhea) indicated the occurrence of moderate OHSS. The presence of clinically evident ascites and/or hydrothorax, increased blood viscosity and coagulation abnormality indicated the occurrence of severe OHSS. Patients with severe OHSS were hospitalized. OHSS with an onset ≤ 10 days from oocyte retrieval was defined as early onset OHSS and OHSS with later onset was defined as late onset OHSS.

Clinical and ultrasound examinations were performed on the day of embryo transfer and after 4, 8 and 14 days to detect the occurrence of OHSS. All patients were instructed to contact us if they experienced difficulty in breathing, decreased urine volume, dizziness on standing, abdominal pain, enlargement of the abdomen and rapid weight gain.

The primary outcome measure was the incidence of moderate/severe OHSS and the secondary outcome measures were the number of retrieved oocytes, metaphase II oocytes, fertilization rate, implantation rate, miscarriage rate, clinical pregnancy rate (presence of intrauterine gestational sac detected by transvaginal ultrasound), and ongoing pregnancy rate (pregnancies continued beyond 20 weeks).

### Sample size calculation

At the time of study design, a Cochrane review revealed that the incidence of moderate or severe OHSS in patients at high risk of developing OHSS treated with cabergoline was 16.95 % [2]. We considered that 11.95 % difference in the incidence of moderate/severe OHSS between the group of patients treated with cabergoline alone and the group of patients treated with the antagonist rescue protocol combined with cabergoline would be of clinical significance. To detect an 11.95 % difference in the incidence of moderate/severe OHSS between both groups, 107 patients would have to be recruited to each group of the study to achieve a study power of 80 % at a significance level of 0.05 (calculated on https//www.sealedenvelope.com/power/continuous-superiority). We expected that the dropout incidence would be 10 %; therefore, 118 patients were recruited to each arm of the study.

### Statistical analysis

Statistical analysis was performed using student’s t test and χ2 test, as appropriate. Fisher’s exact test was used when the expected frequency was less than 5. A probability value (*P* value) less than 0.05 was considered statistically significant. All statistical calculations were performed using Excel version 7 (Microsoft, New York, NY, USA) and SPSS (SPSS, Chicago, IL, USA). Absolute risk reduction (95 % CI) and number needed to treat (95 % CI) were calculated on http://graphpad.com/quickcalcs/NNT1/. An intention to treat analysis and per protocol analysis were performed.

## Results

**Fig. 1:**
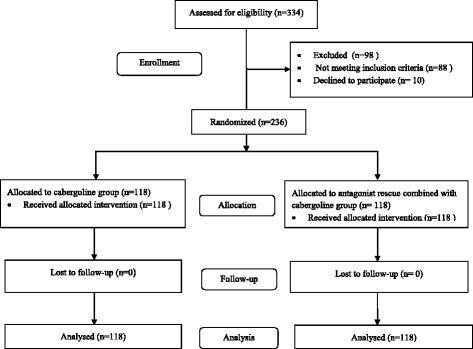
The flow of the patients in the study

Between March 2013 and December 2015, 2458 IVF-ET cycles were performed in our institution. Among the 334 patients who were at high risk for OHSS, 88 patients did not meet the inclusion criteria and 10 patients declined to participate in the study. We enrolled 236 patients with 118 patients randomized to each arm of the study. The flow of the patients in the study is shown in Fig. 1.

There were no significant differences between both groups with respect to age, body mass index (BMI), duration of infertility, cause of infertility, antral follicle count and baseline hormonal profile (Table 1).

**Table 1:** Baseline characteristics of patients

	Cabergoline group (*n* = 118)	Antagonist rescue combined with cabergoline group (*n* = 118)	*P* value
Age (years)	27.35 ± 5.98	26.69 ± 5.58	0.387
Body mass index (Kg/m^2^)	25.85 ± 4.54	26.47 ± 4.23	0.28
Duration of infertility (years)	5.76 ± 2.65	6.14 ± 2.58	0.275
Type of infertility			
• Primary	102/118(86.44 %)	97/118(82.2 %)	0.371
• Secondary	16/118(13.56 %)	21/118(17.8 %)	0.371
Basal FSH (IU/L)	6.29 ± 1.53	6.31 ± 1.48	0.897
Antral follicle count	21.46 ± 4.17	22.05 ± 3.75	0.252
Patients with amenorrhea or oligomenorrhea	12/118(10.17 %)	14/118(11.86 %)	0.678
Cause of infertility			
• Tubal Factor	31/118(26.27 %)	40/118(33.9 %)	0.202
• PCOS	32/118(27.12 %)	29/118(24.58 %)	0.656
• Unexplained	17/118(14.41 %)	12/118(10.17 %)	0.322
• Male Factor	20/118(16.95 %)	22/118(18.64 %)	0.734
• Two or more factors	18/118(15.25 %)	15/118(12.71 %)	0.573

*PCOS* polycystic ovary syndrome

Values are expressed as mean ± SD or n/n(%)

The serum estradiol level on the day of HCG administration was significantly higher in the cabergoline group than in the antagonist rescue combined with cabergoline group (5501 ± 1122 Vs. 2811 ± 399 pg/ml, *P* value < 0.001) (Table 2).

**Table 2:** Outcome of ovarian stimulation

	Cabergoline group (*n* = 118)	Antagonist rescue combined with cabergoline group (*n* = 118)	*P* value
Duration of HP-uFSH Stimulation (days)	9.92 ± 0.85	10.08 ± 0.68	0.129
Total HP-uFSH ampoules (75U)	29.88 ± 6.8	27.87 ± 5.58	0.014
Estradiol level on the HCG day (pg/ml)	5501 ± 1122	2811 ± 399	<0.001
No. of retrieved oocytes	19.09 ± 4.01	18.85 ± 4.37	0.651
No. of metaphase II oocytes	17.02 ± 3.8	16.97 ± 3.94	0.933
No. of fertilized oocytes	13.69 ± 3.32	13.42 ± 3.08	0.523
Fertilization rate	1584/2003(79.08 %)	1561/1940(80.46 %)	0.28
No. of high quality embryos	4.02 ± 1.28	3.84 ± 1.31	0.295
No. of embryos transferred	2.32 ± 0.47	2.26 ± 0.44	0.303
Cycles cancelled	4/118(3.39 %)	0/118(0 %)	0.122
Moderate or severe OHSS	16/118(13.56 %)	6/118(5.08 %)	0.025
Moderate OHSS	12/118(10.17 %)	6/118(5.08 %)	0.141
Severe OHSS	4/118(3.39 %)	0/118(0 %)	0.122
Early onset OHSS	12/118(10.17 %)	5/118(4.24 %)	0.078
Late onset OHSS	4/118(3.39 %)	1/118(0.85 %)	0.37

*OHSS* ovarian hyperstimulation syndrome

Values are expressed as mean ± SD or n/n(%)

In the antagonist rescue combined with cabergoline group, the mean (SD) number of antagonist injections needed to reduce the estradiol level below 3500 pg/ml was 1.52(0.69). Seventy patients (59.32 %) required one GnRH antagonist dose, 35 patients (29.66 %) required two GnRH antagonist doses and 13 patients (11.02 %) required three GnRH antagonist doses to reduce the estradiol levels below 3500 pg/ml. The mean serum estradiol dropped by 34.56 % after the first 24 h of GnRH antagonist administration (5101 Vs. 3338 pg/ml). The drop in the serum estradiol level continued after the second and the third GnRH antagonist injections.

The incidence of moderate/severe OHSS was significantly lower in the antagonist rescue combined with cabergoline group [5.08 % Vs 13.56 %, *P* value =0.025, OR = 0.342, 95 % CI,0.129–0.906]. Absolute risk reduction was 8.48 % (95 % CI, 1.14 %–15.81 %) and number needed to treat was 12 (95 % CI, 6.3–88.1). Four patients in the cabergoline group had severe OHSS and none of the patients in the antagonist group had severe OHSS. Four cycles were cancelled in the cabergoline group and no cycles were cancelled in the antagonist rescue combined with cabergoline group (Table 2).

All patients in the antagonist rescue combined with cabergoline group received 75 IU/day of HP-uFSH when the leading follicle reached 16 mm and all patients in the cabergoline group received at least 150 IU/day of HP-uFSH during the stimulation period. The total gonadotropins dose administered till the leading follicle reached 16 mm was comparable between both groups [25.39 ± 5.64 Vs. 24.32 ± 6.62 ampules, P value = 0.184]. Conversely, the total gonadotropins dose administered after the leading follicle reached 16 mm was significantly lower in the antagonist rescue combined with cabergoline group (2.48 ± 0.64 Vs. 5.56 ± 1.92 ampules, *P* value < 0.001).

There were no significant differences in the number of retrieved oocytes, metaphase II oocytes, fertilized oocytes, high quality embryos (grade 1 or 2) and fertilization rate between the two groups (Table 2). Moreover, the implantation rate and clinical and ongoing pregnancy rates were comparable between the two groups (Table 3). A case of ectopic pregnancy was reported in the antagonist rescue combined with cabergoline group.

**Table 3:** Reproductive outcomes

	Cabergoline group (*n* = 118)	Antagonist rescue combined with cabergoline group (*n* = 118)	Odd ratio (95 % CI)	*P* value
Clinical pregnancy/started cycle	42/118(35.59 %)	46/118(38.98 %)	0.87[0.51–1.47]	0.59
Clinical pregnancy/transfer cycle	42/114(36.84 %)	46/118(38.98 %)	0.91[0.54–1.55]	0.74
Ongoing pregnancy/started cycle	37/118(31.36 %)	41/118(34.75 %)	0.86[0.5–1.48]	0.578
Ongoing pregnancy/transfer cycle	37/114(32.46 %)	41/118(34.75 %)	0.9[0.52–1.56]	0.708
Implantation rate	49/265(18.49 %)	53/267(19.85 %)	0.92[0.59–1.41]	0.689
Multiple pregnancy rate	7/42(16.67 %)	6/46(13.04 %)	1.33[0.41–4.34]	0.632
Spontaneous abortion rate	5/42(11.9 %)	5/46(10.87 %)	1.11[0.3–4.14]	0.879

Values are expressed as n/n (%) unless otherwise indicated

## Discussion

The data presented in this study revealed that GnRH antagonist rescue protocol combined with cabergoline minimized the risk of moderate/severe OHSS and prevented cycle cancelation without affecting the number of oocytes retrieved, quality of embryos and implantation and pregnancy rates in patients at high risk for OHSS who were pretreated with GnRH agonist long protocol. To our knowledge, this is the first randomized controlled study that evaluated the role of antagonist rescue protocol combined with cabergoline in minimizing the risk of OHSS.

The presence of a large number of growing follicles and rapidly rising estradiol level are associated with an increased likelihood of developing OHSS. The causative role of estradiol in inducing OHSS has not been proved. It seems that patients with a large number of growing follicle have a large mass of granulosa cells that produce excessive amounts of estradiol and chemical mediators that are responsible for OHSS [14]. Several authors have proposed several strategies, such as coasting and antagonist rescue protocol, to reduce estradiol levels in an effort to minimize the risk of OHSS [8, 9, 15, 16].

Coasting is one of the most commonly used preventive measures for OHSS. With this management strategy, gonadotropins administration is stopped, GnRH agonist administration is continued and HCG administration is delayed until estradiol drops to a safe level (≤3000 pg/ml). Withholding gonadotropins administration induces apoptosis of granulosa cells and therefore minimizes the production of estradiol and chemical mediators responsible for the development of OHSS [15, 16]. Previous studies revealed that the duration of coasting is an important predictor of IVF outcomes. It seems that prolonged complete deprivation of gonadotropins (>3 days) is associated with a decrease in the number of oocytes retrieved, implantation rate and clinical pregnancy rate [17, 18]. Antagonist rescue of agonist IVF cycles at risk of OHSS was first described by Gustafson et al. [8]. In a retrospective study including 87 patients down-regulated with GnRH agonist who were at risk of OHSS, GnRH agonist was switched to GnRH antagonist in patients who failed to response to reducing the dose of gonadotropins. HCG was administered when the serum estradiol level fell to a safe level (<3000 pg/ml). Antagonist rescue resulted in a rapid decline in the serum estradiol levels without adverse effects on embryo quality, the fertilization rate or the pregnancy rates. The incidence of moderate/severe OHSS was 4.6 % [8].

The exact mechanism by which GnRH antagonists induce a rapid decline in the estradiol level and reduce the risk of OHSS is not clear. Several studies have suggested that GnRH antagonists have a direct effect on the ovary mediated by interaction with the GnRH-I and GnRH-II receptors present on granulosa cells [19, 20]. Asimakopoulos et al. [21] reported that GnRH antagonists inhibit the production of VEGF from cultured human granulosa luteinized cells.

In a prospective randomized study, Aboulghar et al. [9] compared coasting with antagonist rescue protocol in a series of 192 patients pretreated with GnRH agonist long protocol who were at high risk for OHSS. None of the patients in either groups experienced severe OHSS. The duration of coasting was significantly longer than the duration of antagonist administration (2.81 ± 0.97 Vs. 1.74 ± 0.91 days; *P* < 0.001). Antagonist rescue protocol produced more high quality embryos and more oocytes. The rapid drop in the estradiol level with antagonist rescue protocol prevented prolonged deprivation of the growing follicles from gonadotropins which has a negative impact on the embryo quality and IVF-ET outcomes. Moreover, the administration of a low dose of gonadotropins (75 IU/day) in the antagonist rescue protocol group supported the function of granulosa cells and maintained the production of high quality embryos. Martinaz et al. [22] replaced GnRH agonist with GnRH antagonist in the early stages of ovarian stimulation (after 6 to 8 days of gonadotropins administration) in a series of 19 patients who were at high risk for OHSS (more than 10 follicles per ovary and/or serum estradiol > 900 pg/ml and the follicles expected to need 3 or more days to reach triggering criteria). The dose of gonadotropins was reduced and ovulation was triggered by rHCG in 14 patients and by a GnRH agonist bolus in 5 patients. The mean (SD) duration of antagonist administration was 4.8 ± 0.8 days. No oocytes were retrieved in one patient triggered with a GnRH agonist bolus. One patient had moderate OHSS. The authors suggested that early administration of GnRH antagonist could allow triggering of ovulation with GnRH agonist bolus which is associated with a lower risk of OHSS compared to triggering ovulation with HCG. Further studies are needed to determine for how long pituitary desensitization and unresponsiveness to GnRH bolus trigger persist after switching GnRH agonist to GnRH antagonist.

In a retrospective study, Hill et al. [23] replaced GnRH agonist with GnRH antagonist without reducing the dose of gonadotropins in 387 patients pretreated with microdose flare or long luteal agonist protocol who were at high risk of OHSS. In the majority of patients (98.45 %), one or two doses of GnRH antagonist were required to induced a rapid drop in the estradiol level. Antagonist rescue prevented cycle cancellation, as well as permitted continued gonadotropin administration and follicular development and maturation for an additional one to three days. The incidence of severe OHSS was 8 % and the cycle cancellation rate was 1.5 %.

The higher incidence of severe OHSS in the study by Hill et al. [23] (8 %) in comparison with our study (0 %) and the studies of Aboulghar et al. [9] (0 %), Martinez et al. [22] (0 %) and Gustafson et al. [8] (2.35 %) may be attributed to differences in the sample population or the treatment protocol. In the study by Hill et al. [23], antagonist administration was not accompanied with adjustment in the dose of gonadotropins, whereas the dose of gonadotropins was reduced in other studies [8, 9, 22]. Moreover, the duration of antagonist administration was longer in Martinaz et al. [22] study in comparison with the study by Hill et al. [23]. Further studies are needed to investigate the proper timing and duration of antagonist administration and the effect of reducing gonadotropins dose on the incidence of OHSS, the number of retrieved oocytes and the embryo quality.

Several studies revealed that the incidence of OHSS is lower in mild stimulation IVF cycles in comparison to conventional long agonist IVF cycles [24, 25]. Reducing gonadotropins dose seems to be effective in minimizing the risk of OHSS. Large follicles have higher sensitivity to gonadotropins compared to medium and small sized follicles, and therefore with reducing the dose of gonadotropins, large follicles continue to grow and small and medium sized follicles become atretic. The atresia of medium and small sized follicles minimizes the production of chemical mediators responsible for development of OHSS [26]. Moreover, reducing gonadotropins dose slows the rate of granulosa cells proliferation which produces the chemical mediators responsible for OHSS [27].

Antagonist rescue protocol and cabergoline minimize the risk of OHSS by two different mechanisms. Antagonist rescue protocol inhibits estradiol and VEGF production by the growing follicles and cabergoline blocks VEGF mediated increased capillary permeability [2, 21]. This means that cabergoline augments the effect antagonist rescue protocol.

In the current study, 5000 IU of HCG was used to trigger final oocyte maturation. The majority of studies revealed that the reproductive outcomes of IVF-ET cycles are comparable in women receiving 5000 IU or 10000 IU of HCG to trigger final oocyte maturation [28]. Several authors suggested that the administration of 5000 IU of HCG instead of 10000 IU of HCG may minimize the risk of OHSS in patients who have a high risk for OHSS [28, 29]. On the other hand, two small studies revealed that the administration of lower doses of HCG (≤5000 IU) does not reduce the incidence of OHSS in patients at high risk for OHSS [30, 31].

In the current study, the administration of 5000 IU of HCG instead of 10000 IU of HCG to trigger final oocyte maturation may have an additive effect in minimizing the risk of OHSS in both groups. However, because the same dose of HCG (5000 IU) was used to trigger final oocyte maturation in both groups, we do not think that the administration of 5000 IU of HCG instead of 10000 IU of HCG is responsible for the greater efficacy of antagonist rescue protocol combined with cabergoline in minimizing the risk of OHSS in comparison to cabergoline alone.

The main strength of this study is the prospective randomized controlled design. Our study has two limitations. First, the patients and the doctors were not blind to the treatment allocation. Second, it is not clear whether antagonist administration, HP-uFSH dose reduction to 75 IU/day or the combination of both is responsible for minimizing the risk of OHSS. Further studies are needed to clarify this point.

## Conclusion

GnRH antagonist rescue protocol (replacing GnRH agonist with GnRH antagonist and reducing the dose of gonadotropins to 75 IU/day) combined with cabergoline is more effective than cabergoline alone in the prevention of OHSS in patients downregulated with GnRH agonist who are at high risk for OHSS.

### Ethics approval and consent to participate

The ethics committee of Aljazeera (Al Gazeera) hospital approved the study protocol [N-2-2013] and the patients provided informed consent before randomization.

## Abbreviations

BMI: body mass index
FSH: follicle stimulating hormone
GnRH: gonadotropin releasing hormone
HCG: human chorionic gonadotropin
HP-uFSH: highly purified urinary follicle stimulating hormone
IVF-ET: in vitro fertilization embryo transfer
OHSS: ovarian hyperstimulation syndrome
PCOS: polycystic ovary syndrome
rHCG: recombinant human chorionic gonadotropins
TNF: tumor necrosis factor
VEGF: vascular endothelial growth factor
